# Short-term memory impairment following recovery from systemic inflammation induced by lipopolysaccharide in mice

**DOI:** 10.3389/fnins.2023.1273039

**Published:** 2023-10-18

**Authors:** Kohei Morimoto, Shu Watanuki, Ryota Eguchi, Taisuke Kitano, Ken-ichi Otsuguro

**Affiliations:** ^1^Laboratory of Pharmacology, Department of Basic Veterinary Sciences, Faculty of Veterinary Medicine, Hokkaido University, Sapporo, Japan; ^2^Laboratory of Veterinary Biochemistry, School of Veterinary Medicine, Kitasato University, Aomori, Japan

**Keywords:** lipopolysaccharide, neuroinflammation, dendritic spine, memory, astrocyte, microglia, hippocampus, gliosis

## Abstract

The relationship between neuroinflammation and mental disorders has been recognized and investigated for over 30 years. Diseases of systemic or peripheral inflammation, such as sepsis, peritonitis, and infection, are associated with increased risk of mental disorders with neuroinflammation. To elucidate the pathogenesis, systemic administration of lipopolysaccharide (LPS) in mice is often used. LPS-injected mice exhibit behavioral abnormalities with glial activation. However, these studies are unlikely to recapitulate the clinical pathophysiology of human patients, as most studies focus on the acute inflammatory response with systemic symptoms occurring within 24 h of LPS injection. In this study, we focus on the effects of LPS on behavioral abnormalities following recovery from systemic symptoms and investigate the mechanisms of pathogenesis. Several behavioral tests were performed in LPS-injected mice, and to assess neuroinflammation, the time course of the morphological change and expression of inflammatory factors in neurons, astrocytes, and microglia were investigated. At 7 days post-LPS injection, mice exhibited short-term memory impairment accompanied by the suppression of neuronal activity and increases in morphologically immature spines. Glial cells were transiently activated in the hippocampus concomitant with upregulation of the microglial phagocytosis marker CD68 3 days after injection. Here we show that transient glial cell activation in the acute response phase affects neuronal activity and behavior following recovery from systemic symptoms. These findings provide novel insights for studies using the LPS-induced inflammation model and that will contribute to the development of treatments for mental disorders of this etiology.

## Graphical abstract

Pathological conditions of short-term memory impairment with hippocampal neuroinflammation in LPS-injected mice

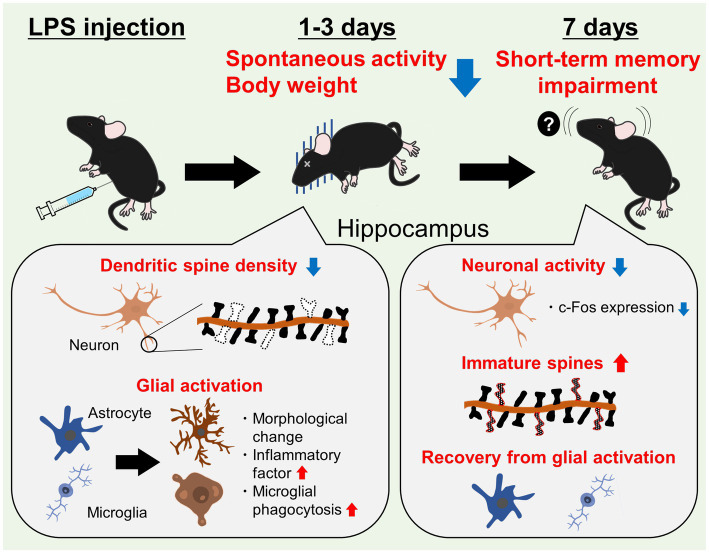

## 1. Introduction

Mental disorders have become a major public health burden in many countries and are estimated to cause a loss of US $16 trillion over 20 years in the global economy (Patel et al., 2018). The monoamine hypothesis has been considered to be an underlying mechanism of mental disorders for more than half a century (Hirschfeld, 2000). Antidepressants and antipsychotics have been developed based on this hypothesis, but many patients are refractory to treatments targeting this regulatory mechanism, supporting the likelihood of additional regulatory mechanisms such as the neuroplasticity and neurogenesis hypotheses (Boku et al., 2018). The relationship between neuroinflammation and mental disorders has been recognized and investigated for over 30 years (Maes et al., 1992; Roohi et al., 2021). Clinical studies have demonstrated that patients with systemic or peripheral inflammatory diseases such as sepsis, enteritis, periodontitis, and infection are at increased risk for mental disorders such as major depressive disorder, dementia, schizophrenia, and Alzheimer’s disease (Arias et al., 2012; Davydow et al., 2013; Choi et al., 2019; Sharma et al., 2021). In addition, meta-analyses have revealed that brain and blood levels of inflammatory factors are increased in patients with mental disorders (Yuan et al., 2019; Dunn et al., 2020).

To elucidate the pathogenesis of these debilitating diseases, several rodent models of inflammation-induced mental disorders have been developed (Nazem et al., 2015; Na and Krishnamoorthy, 2021; Tamura et al., 2022). Among these models, systemic administration of lipopolysaccharide (LPS), a bacterial toxin, is often used, and LPS-injected mice exhibit abnormalities such as depressive-, anxiety-, and cognitive disorder-like behavior with neuroinflammation in some contexts (Song and Wang, 2011; Hoogland et al., 2015). Acute glial cell activation with morphological changes is observed within 24 h of LPS injection (Bowyer et al., 2020), and plays a central role in neuroinflammation. Several studies reveal that microglia, the brain’s resident innate immune cells, are activated by LPS, resulting in increased production of inflammatory cytokines and enhanced phagocytosis (Fricker et al., 2012; Lively and Schlichter, 2018; Jiang et al., 2022). Additionally, reactive astrocytes response to LPS increase permeability of the blood–brain barrier (BBB) triggering a more drastic neuroinflammation, and change the balance between release and uptake of neurotransmitters in tripartite synapses (Pascual et al., 2012; Peng et al., 2021). Astrocytes can also release inflammatory cytokines, as prior reports, including our own, have demonstrated astrocytic cytokine production (Morimoto et al., 2020, 2021, 2022). Activated glial cells can accelerate neurodegeneration, resulting in functional impairment *via* disturbed synaptic plasticity, neural connectivity, and neurotransmitter balance (Almeida et al., 2020; Bennett and Viaene, 2021). Therefore, it is important to elucidate and control the pathogenesis of LPS-induced neuroinflammation, especially glial cell activation, for the treatment development.

However, these studies using the LPS model are unlikely to recapitulate the clinical pathophysiology of human patients, as most studies focus on the acute inflammatory response occurring within 24 h of LPS injection (Yin et al., 2023). This compromises clinical relevance for two reasons: the timepoint of previous studies using the LPS model is unlikely to be consistent with human disease progression; for example, clinical sepsis and peritonitis patients develop cognitive dysfunction after recovery from acute symptoms (Hernandez-Ruiz et al., 2022; Li et al., 2022). Secondly, the results of behavioral tests conducted within 24 h after LPS injection could be affected by both aberrations in brain function and systemic deficits, as significant systemic symptoms can occur in these rodent models. Therefore, behavioral tests should be conducted at least a few days following LPS challenge, after recovery from systemic and acute symptoms, and the time course of neuroinflammatory conditions during the period should also be investigated.

In the present study, we conducted several behavioral tests for the purpose of elucidating behavioral abnormalities in LPS-injected mice following recovery from systemic and acute symptoms. We subsequently investigated the hippocampal CA1 region, which is associated with short-term memory, and comprehensively investigated the pathological sequalae of neurons, astrocytes, and microglia to elucidate the underlying regulatory mechanisms for the pathogenesis.

## 2. Materials and methods

### 2.1. Animals

Prior to initiating studies, all animal care and experimental protocols were approved by the Committee on Animal Experimentation, Faculty of Veterinary Medicine, Hokkaido University (No. 19–0079), which was awarded Accreditation Status by the Association for Assessment and Accreditation of Laboratory Animal Care International (AAALAC International). C57BL/6 N mice were obtained from CLEA Japan (Tokyo, Japan). All mice were maintained at 22 ± 4°C under a 12/12 h light/dark cycle (7 am–7 pm) with *ad libitum* food and water access. Male mice (11–14 weeks) were used for experiments.

### 2.2. LPS treatment

LPS (#L2630, *Escherichia coli* O111:B4) was purchased from Sigma-Aldrich (St. Louis, MO, United States). A stock solution (10 mg/mL) was prepared in distilled water and stored at −20°C until injection. Mice received a single-dose intraperitoneal (*i.p.*) injection of LPS (1 or 3 mg/kg body weight) in saline (10 mL/kg body weight) at 7 pm (at the end of the light cycle). Mice were used for each experiment after the recovery periods outlined for specific experiments described as on days 0–7.

### 2.3. Behavioral tests

All behavioral tests and measurements of body weight and water intake were conducted at 7 pm. We selected seven behavioral tests commonly used in LPS-injected mouse models to evaluate brain functions and abnormalities such as spontaneous activity, anhedonia, anxiety-, depression-like behavior, and memory (Yin et al., 2023). In all behavioral tests, all apparatuses were wiped with 70% EtOH after each mouse use to prevent smell from affecting animal behavior.

#### 2.3.1. Open field test

Mice were placed in the center of an open field apparatus (45 cm × 45 cm × 45 cm) and allowed to explore freely for 10 min. All areas of the open field were recorded using a digital camera and behavioral analysis was performed automatically using the Mouse Behavioral Analysis Toolbox (MouBeAT) as described (Bello-Arroyo et al., 2018). Total distance traveled was used as an evaluation of spontaneous activity, and total freezing time and time spent in the center (27 cm × 27 cm) were used as an evaluation of anxiety-like behavior (Sestakova et al., 2013).

#### 2.3.2. Sucrose preference test

Two drinking water bottles were placed in the home cages and made freely accessible to the mice. For a 3 day habituation period, both bottles were filled with 1% sucrose for the first day, and for the subsequent 2 days, one bottle was filled with 1% sucrose and the other with distilled water. LPS was injected after the 3 day habituation, and intake of 1% sucrose and distilled water were measured every 24 h. Bottle locations were switched daily to avoid intake depending on position. The percentage of 1% sucrose intake relative to total water intake was used as an indicator of anhedonia (Liu et al., 2018).

#### 2.3.3. Y-maze test

Mice were placed in a Y-shape apparatus consisting of three arms (length 30 cm, width 6 cm) and allowed to freely move among the three arms. Alternation scores were measured as described previously (Miedel et al., 2017), using the number of total arm entries and the number of three consecutive entries into different arms. Alternation scores were evaluated automatically using MouBeAT software and considered an indicator of working memory (Kraeuter et al., 2019).

#### 2.3.4. Forced swimming test

A 3,000 mL plastic beaker (diameter 15 cm) was filled with water (25°C, height 23 cm) to a level that prevented mice from touching the bottom. Mice were placed in the beaker for 6 min. Immobility time, defined as the time that the mouse’s head and limbs did not move, was used as an indicator of depression-like behavior (Yankelevitch-Yahav et al., 2015). Immobility time was measured visually by human scorers during the last 5 min of the test.

#### 2.3.5. Elevated plus maze test

Mice were placed in the center of an apparatus consisting of two open arms (length 30 cm, width 6 cm), a central platform (6 cm × 6 cm), and two closed arms (30 cm × 6 cm). The closed arms were enclosed by walls (height 30 cm) and located 63 cm above the floor. Mice were allowed to move freely for 10 min. All areas of the maze were recorded using a digital camera and behavioral analysis was performed automatically using MouBeAT. The time spent in each arm was measured, and the time spent in the closed arm was used as an indicator of anxiety-like behavior (Sestakova et al., 2013).

#### 2.3.6. Novel object recognition test

The novel object recognition test (NORT) consisted of three stages: habituation, training, and testing, and was conducted 5, 6, and 7 days after LPS injection. An open field (30 cm × 30 cm) with no objects in the habituation section, a field with two identical objects in the training section, and a field with one of the objects replaced by a novel object in the test section, as described previously (Leger et al., 2013). Mice were allowed to move freely in these fields for 10 min. A T25 flask was used as the original object and a 50 mL tube was used as the novel object. Mice were tested to ensure that there was no difference in potential preference between the two objects (Supplementary Figure S1A). The times that the mouse spent actively exploring each object, defined as having its nose pointed at the object, were used as the exploration time. In the test section, the time difference was determined from the time the mouse was placed in the field to the time it first explored each object (original object - novel object (s)). The exploration time and the time difference were evaluated automatically using the MouBeAT and used as an indicator of short-term memory (Chesworth et al., 2018).

#### 2.3.7. Object location recognition test

The object location recognition test (OLRT) was identical to NORT until the training section, in which one of the objects is moved to a new location (opposite corner) instead of being replaced by a novel object in the test section. The exploration time and the time difference were evaluated automatically using the MouBeAT and used as an indicator of short-term memory (Chesworth et al., 2018).

### 2.4. Endotoxin assay

A ToxinSensor™ Chromogenic LAL Endotoxin Assay Kit (GenScript, NJ, United States) was used to quantify endotoxin levels of the plasma and brain. Mice were anesthetized by inhalation of isoflurane (Pfizer, New York, NY, United States). Blood samples were taken from anesthetized animals by direct cardiac stick and mixed with EDTA. After perfusion with 10 mL saline, the whole brain was isolated, weighed, and homogenized in 1 mL distilled water. Blood and brain samples were centrifuged at 1,500 × g, and supernatants were used as samples. Endotoxin levels were quantified according to manufacturer’s instructions. All equipment used for the experiment was endotoxin-free. Endotoxin levels were quantified as endotoxin units (EU) in plasma volume (EU/mL) and as EU per brain wet weight (EU/g).

### 2.5. Evaluation of blood–brain barrier permeability

BBB permeability was evaluated using Evans blue dye, which binds to albumin in blood, as described (Manaenko et al., 2011). Evans blue (2% w/v) was injected *i.p.* (4 mL/kg body weight), and 3 h later, mice were anesthetized by inhalation of isoflurane and perfused with 10 mL saline. Then, the whole brain was isolated, weighed, and homogenized in 100% trichloroacetic acid. The brain sample was incubated at 4°C for at least 12 h and then centrifuged at 500 × g, and the supernatants were used for measurement of absorbance at 610 nm using a microplate reader (SH-1000; Corona Electric, Hitachinaka, Japan). Brain Evans blue amounts were calculated using a standard curve and normalized to brain wet weight (g Evans blue/g wet brain weight). Brain dry weight was measured after the wet brain was incubated at 60°C for at least 12 h. The water content (%) was determined from the difference in the wet and dry weights divided by the wet weight.

### 2.6. Immunohistochemistry

Mice were anesthetized by inhalation of isoflurane and perfused with 4% paraformaldehyde (PFA) in phosphate-buffered saline (PBS). Whole brains were isolated and fixed with 4% PFA overnight at 4°C. Brains were then glued to a slicer stage (LinearSlicer Pro7, Dosaka EM, Kyoto, Japan), flooded in PBS, and cut into 50 μm coronal slices. Slices were blocked with a blocking buffer composed of 10% goat serum, 0.5% Triton X-100, and 0.05% sodium azide in PBS at room temperature (RT) for 2 h. Slices were incubated with primary antibodies (Supplementary Table S1) at 4°C for at least 12 h, and then with Alexa Fluor 488- (#A11008, 1:500, Thermo Fisher Scientific, MA, United States) and/or 555- (#A21422, 1:500) conjugated goat anti-rabbit or mouse antibody at RT for 90 min. Slices were mounted onto glass slides with DAPI-Fluoromount G (SouthernBiotech, Birmingham, AL, United States). Images were captured using a laser scanning confocal microscope (LSM 700, Carl Zeiss, Oberkochen, Germany) with a 40 × lens objective. The images were comprised of 30 μm Z-stacks consisting of 31 optical slices of 1 μm thickness by maximum intensity projection. The mean intensity or areas of GFAP, Iba-1, and CD68 were analyzed by ImageJ software (National Institutes of Health, MD, United States) and normalized to the control, which was arbitrarily set to a value of “1.0.”

### 2.7. Staining of degenerating neurons

PathoGreen™ Histofluorescent Stain (Biotium, CA, United States) was used to detect neuronal degeneration. Experiments were performed according to manufacturer’s instructions. Briefly, the brain was perfused and fixed with 4% PFA overnight at 4°C. Then, the brain was cut into 50 μm coronal slices, which were dried at 60°C for 30 min. Slices were incubated with 0.06% KMnO_4_ and 1,000 × PathoGreen™ stock solution at RT for 10 min each. After air drying, slices were incubated with xylene and mounted onto glass slides. Images were captured using a laser scanning confocal microscope with a 40 × lens objective.

### 2.8. Golgi-cox staining

Golgi-Cox staining was performed as described previously, but with partial modification (Bayram-Weston et al., 2016). The brain was perfused and fixed with 4% PFA overnight at 4°C, and incubated with a solution consisting of 1.04% Hg_2_Cl_2_, 1.04% K_2_Cr_2_O_7_, and 0.83% K_2_CrO_4_ in distilled water at RT for 2 weeks. The brain was incubated with 25% sucrose at RT for 6 h and subsequently cut into 100 μm coronal slices. After air drying, slices were incubated with 28% ammonia for 10 min. Slices were then incubated with xylene and mounted onto glass slides. Images were captured using a fluorescence microscope (BZ-9000, KEYENCE, Osaka, Japan) with a 100 × lens objective. Morphological evaluation of dendritic spines was conducted automatically using the application “Reconstruction^1^” as described (Risher et al., 2014). Five neurons were randomly selected from slices obtained from one mouse, and one dendrite >30 μm was measured per neuron. The average of the dendritic spine values per mouse was used as an independent data point.

### 2.9. Evaluation of glial cell morphology

Morphological features of astrocytes and microglia were assessed using confocal Z-stack images of GFAP and Iba-1 staining, respectively. Astrocytes were analyzed using SMorph as described previously (Sethi et al., 2021), and microglia were analyzed using ImageJ software as described (Young and Morrison, 2018). All glial cells in one image (approximately 60 cells) per mouse were analyzed. Principal component (PC) analysis was performed using the statistical analysis software JMP® 17 (SAS Institute, Inc., Cary, NC, United States) for astrocytic and microglial morphology using the morphological parameters shown in Supplementary Figures S2, S3.

### 2.10. RNA extraction and RT-qPCR analyses

Total RNA was extracted from whole hippocampi using RNAiso Plus (Takara Bio, Tokyo, Japan). To remove genomic DNA and synthesize cDNA, RNA samples were then incubated with qPCR RT Master Mix with gDNA Remover (TOYOBO, Osaka, Japan). Real-time PCR was performed using Thunderbird SYBR qPCR Mix (TOYOBO), oligonucleotides, and the cDNA reaction solution. Primer sequences are provided in Supplementary Table S2. Thermal cycles were performed using an Eco Real-Time PCR System (Illumina, San Diego, CA, United States). Cycling conditions were 95°C for 1 min (initial denaturation), followed by 40 cycles of denaturation (95°C, 15 s), annealing, and extension (63°C, 30 s). RNAs without reverse transcription were used as negative controls to determine if DNA contamination was present, as indicated by lack of amplification by real-time PCR. Melt curve analysis confirmed that only one amplicon was produced by each reaction. The expression levels of factors relative to *Gapdh* were calculated using the ΔΔCq method and experimental results were normalized to the control, which was arbitrarily set to a value of “1.0.”

### 2.11. Data and statistical analysis

All studies were designed to generate groups of equal size using randomization and blinded analysis. Data are expressed as means ± S.E.M. (*n* = number of independent measurements) of at least five independent experiments (biological replicates). Statistical comparisons between two groups were conducted using the unpaired Student’s t-tests. For multiple comparisons, one-way ANOVAs followed by the Dunnett’s test or Tukey’s test were used. Post-hoc tests were performed only if F achieved *p* < 0.05 and there was no significant inhomogeneity of variance. *p* < 0.05 was considered statistically significant. All statistical analysis was performed using the statistical analysis software JMP® 17.

## 3. Results

### 3.1. LPS caused systemic symptoms and behavioral abnormalities 24 h after injection

**Figure 1:**
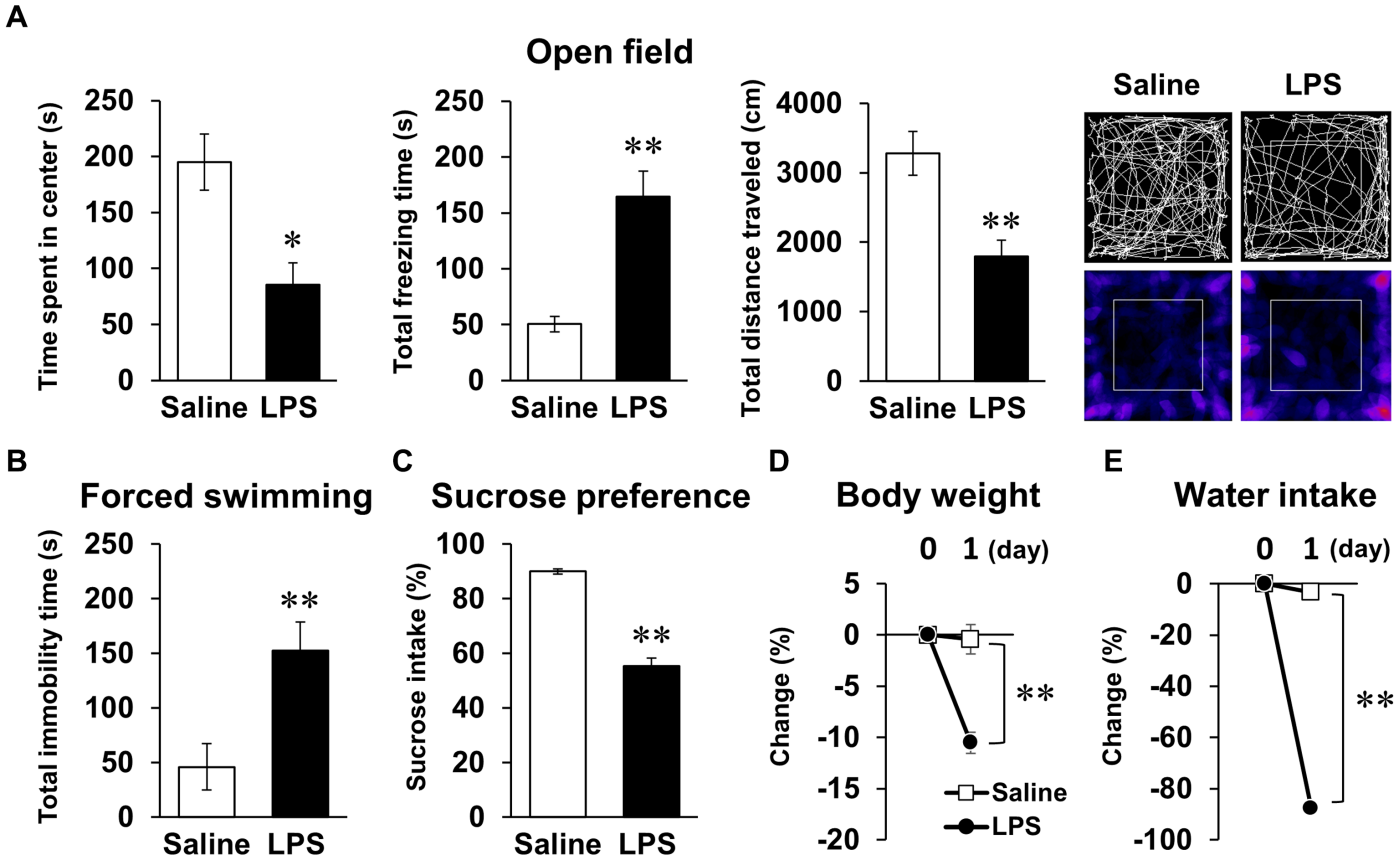
Systemic mouse symptoms and behavioral abnormalities 24 h following LPS injection. **(A)** Time spent in center (s), total freezing time (s), total distance traveled (cm), and representative image of mouse trace and heat map in open field test (10 min) with mice 24 h following LPS injection (1 mg/kg, *i.p.*, single dose). *n* = 5 mice/group. **(B)** Total immobility time (s) in forced swimming test (5 min) in mice 24 h after LPS injection. *n* = 5 mice/group. **(C)** Ratio of sucrose intake (%) over 24 h following LPS injection in sucrose preference test. *n* = 6 mice/group. **(D,E)** Change ratio (%) of body weight **(D)** and water intake **(E)** in mice 24 h after LPS injection. n = 6 mice/group. ^*^p < 0.05, ^**^p < 0.01 (unpaired Student’s t-test). All data are expressed as means ± S.E.M.

We first conducted behavioral tests in mice 24 h after LPS injection, as reported in many previous studies. LPS injection (1 mg/kg*, i.p.*) decreased time spent in center, increased total freezing time in the open field test, and increased total immobility time in the forced swimming test (Figures 1A,B). In addition, sucrose preference decreased during the first 24 h following LPS injection (Figure 1C), but notably, body weight and water intake decreased significantly (Figures 1D,E). Total distance traveled in the open field test, an indicator of spontaneous activity, also decreased (Figure 1A). These results were indicative that the anxiety- and depression-like behavior observed in these behavioral tests could reflect the effects of systemic symptoms.

### 3.2. LPS caused short-term memory impairment after recovery from systemic symptoms 7 days after injection

**Figure 2:**
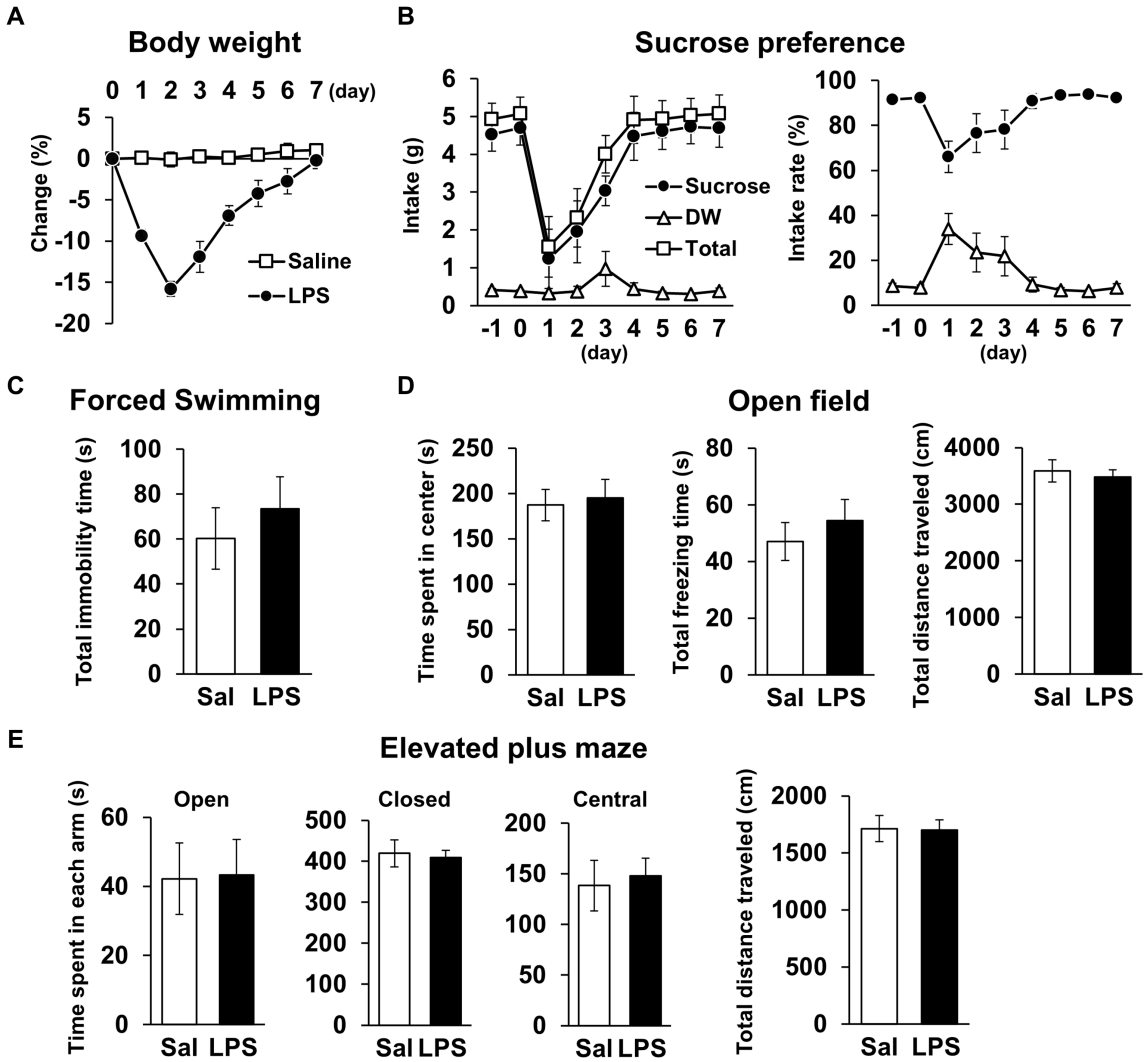
General locomotor activity levels and depression/anxiety-like behavior 7 days after LPS injection. **(A)** Change ratio (%) of body weight 0–7 days after LPS injection (3 mg/kg, *i.p.*, single dose). n = 8 mice/group. **(B)** Weight (g, left) and ratio (%, right) of sucrose intake or distilled water every 24 h with mice −1 − 7 days after LPS injection in sucrose preference test. *n* = 5 mice/group. **(C–E)** Total immobility time (s) in forced swimming test (5 min, **C**), time spent in center (s), total freezing time (s), and total distance traveled **(cm)** in open field test (10 min, **D**), and time spent in each arm (s) and total distance traveled (cm) in elevated plus maze test (10 min, **E**) 7 days after LPS injection. Sal: saline. *n* = 6 mice/group. All data are expressed as means ± S.E.M.

**Figure 3:**
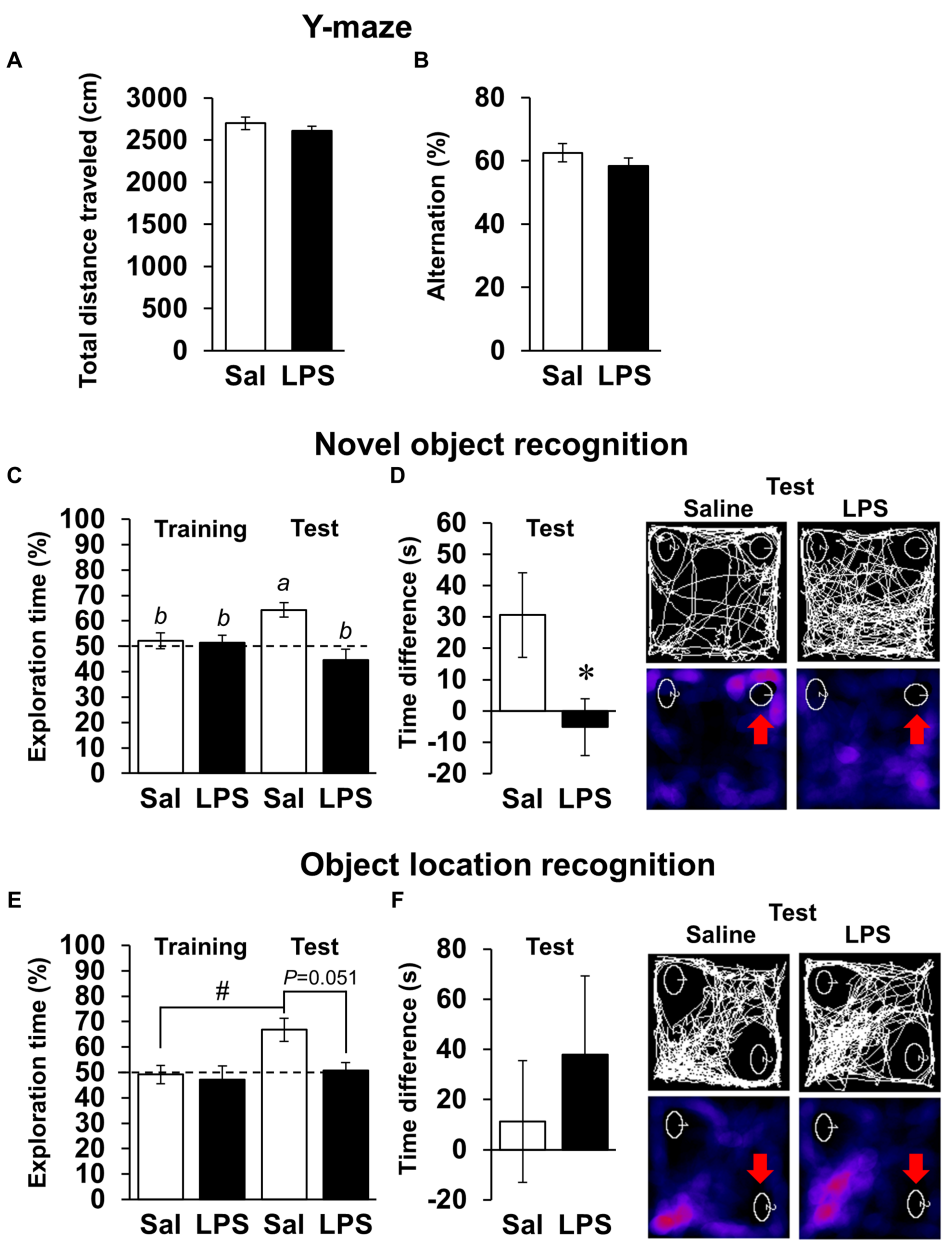
Effects of LPS on working memory and short-term memory 7 days after injection. **(A,B)** Total distance traveled (cm, **A**), alternation score (%, **B**), and representative image of mouse traces in Y-maze tests (8 min) 7 days after LPS injection (3 mg/kg, *i.p.*, single dose). Sal: saline. *n* = 8 mice/group. **(C–F)** Difference in first recognition time (s), exploration time of novel or moved object (%), and representative images of mouse traces and heat maps in novel object recognition tests **(C,D)** or object location recognition tests **(E,F)** 7 days after LPS injection. Red arrow: novel or moved object. *n* = 9–10 mice/group. ^*^p < 0.05 (unpaired Student’s *t*-test). Means with the different letter are significantly different and with the same letter are not significantly different from each other (Tukey’s test, **C**), ^#^*p* < 0.05 (Tukey’s test, **E**). All data are expressed as means ± S.E.M.

Next, we examined whether mice had recovered from systemic symptoms 7 days after LPS injection (3 mg/kg, *i.p*.). Body weight and water intake decreased after LPS injection, which was lowest on day 1 or 2 but recovered by day 7 (Figures 2A,B), and LPS injection 7 days prior did not affect the total distance traveled in any behavioral tests (Figures 2D,E, 3A; Supplementary Figures S1B,C). These results suggested that LPS-injected mice recovered from systemic symptoms on day 7. We then conducted multiple behavioral tests on day 7. LPS injection 7 days prior did not affect the sucrose preference test, forced swimming test, open field test, or elevated plus maze test, which were conducted to evaluate anhedonia-, depression-, and anxiety-behaviors (Figures 2C–E). Prior LPS injection also did not affect the results of the Y-maze test, which was conducted to evaluate working memory (Figure 3B). Next, NORT and OLRT were conducted to evaluate short-term memory. In NORT, the control group exhibited a significant increase in the percentage of exploration time of the novel object in the test section, compared with that of the original object in the training section (Figure 3C). Meanwhile, the LPS group exhibited no increase in the percentage between original and novel objects. There was also a significant difference in the percentage between the control and LPS groups in the test section. Furthermore, we measured the time difference between the first exploration of the novel object and the original object by mice in the test section. The control group explored the novel object earlier than the original object, but the LPS group hardly exhibited any difference between the time to explore each object (Figure 3D). In OLRT, the percentage of exploration time increased for the moved object in the control group, but not in the LPS group (Figure 3E). There was no difference in object recognition time between the two groups (Figure 3F). These results suggest that short-term memory impairment was present on day 7 following LPS injection, after recovery from systemic symptoms.

### 3.3. LPS suppressed neuronal activity accompanied by increased hippocampal immature dendritic spines

**Figure 4:**
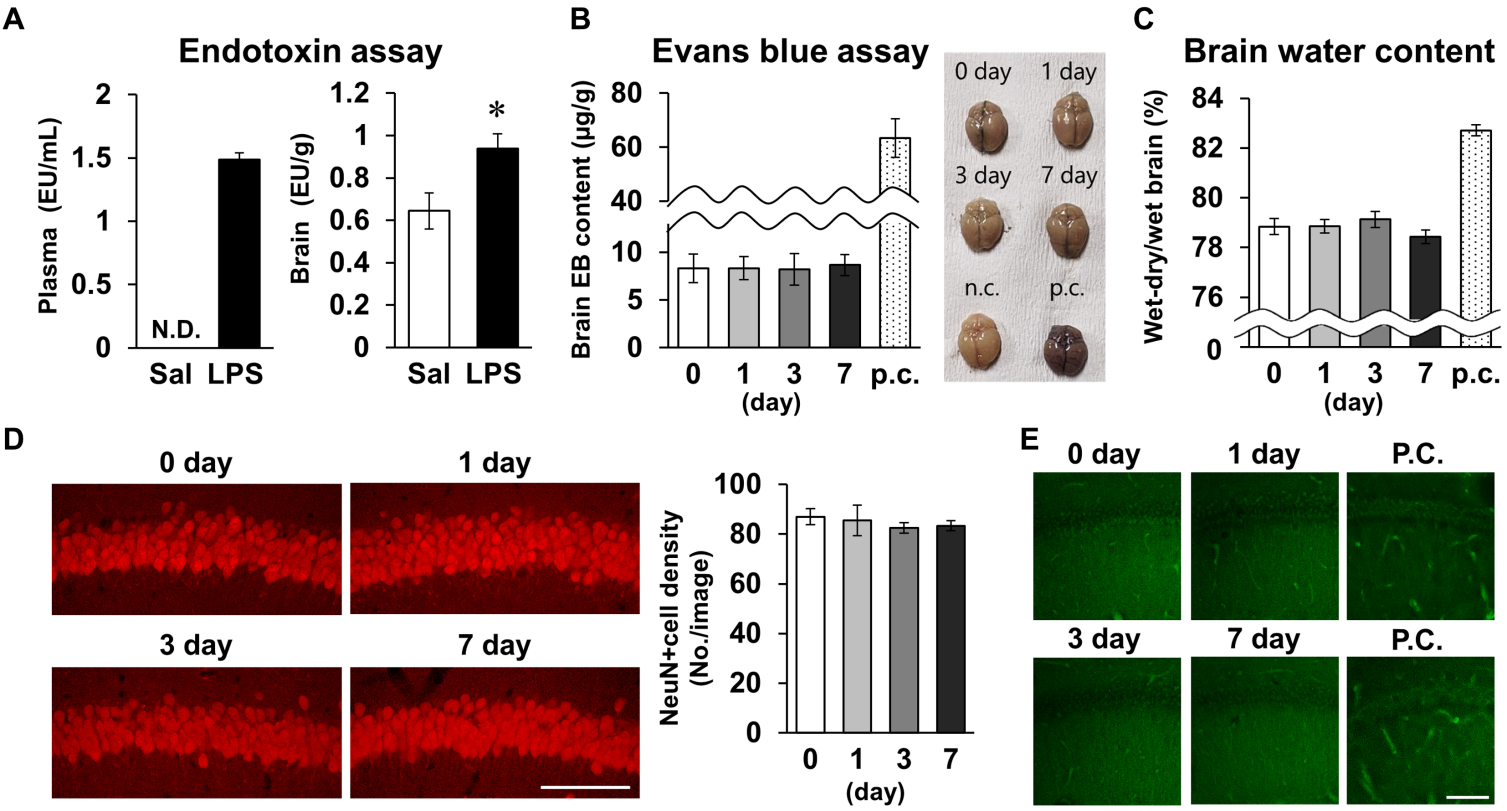
Effects of LPS on blood–brain barrier permeability and neuronal survival. **(A)** Endotoxin level detected by endotoxin assay (LAL tests) of plasma (EU/mL, left) and brain (EU/g, right) tissues of mice 24 h after LPS injection (3 mg/kg, *i.p.*, single dose). *n* = 5 mice/group. ^*^p < 0.05 (unpaired Student’s *t*-test). **(B,C)** Evans blue content (μg/g) and brain representative images **(B)**, and whole-brain water content (wet-dry/wet, %, **C**) 0–7 days after LPS injection. p.c.: positive control (LPS 18 mg/kg, *i.p.*, 18 h). *n* = 5 mice/group. **(D)** Representative NeuN-stained images of hippocampal CA1 regions 0–7 days after LPS injection (left) and NeuN-positive cell images (number of cells/image, right). Scale bar = 100 μm. *n* = 6 mice/group. **(E)** Representative images of the hippocampal CA1 region stained with PathoGreen™. p.c.: positive control (LPS 18 mg/kg, *i.p.*, 18 h). Scale bar = 100 μm. All data are expressed as means ± S.E.M.

As short-term memory impairment was detected on day 7 post-LPS, multiple pathologies were investigated. Plasma LPS level increased to 1.5 ± 0.1 EU/mL 24 h after LPS *i.p.* injection (Figure 4A). Brain LPS levels were also significantly increased 24 h after LPS injection. Because this finding was suggestive of BBB breakdown, we evaluated BBB permeability by measuring migration of Evans blue into the brain and measuring brain water content as an indicator of cerebral edema. Neither index changed significantly on days 0, 1, 3, or 7 post-LPS injection (Figures 4B,C), suggesting that permeability of the BBB did not change. To evaluate neuronal survival in the CA1 area of the hippocampus, which is associated with short-term memory, the density of NeuN-positive cells was quantified, and neuronal cell death was detected by PathoGreen™ staining. The densities of live and dead neurons were not changed on days 0, 1, 3, and 7 post-LPS injection (Figures 4D,E). Based on these results, the experimental conditions of LPS injection used in this study increased the amount of toxin in the blood and brain, but did not affect the BBB or neuronal survival.

**Figure 5:**
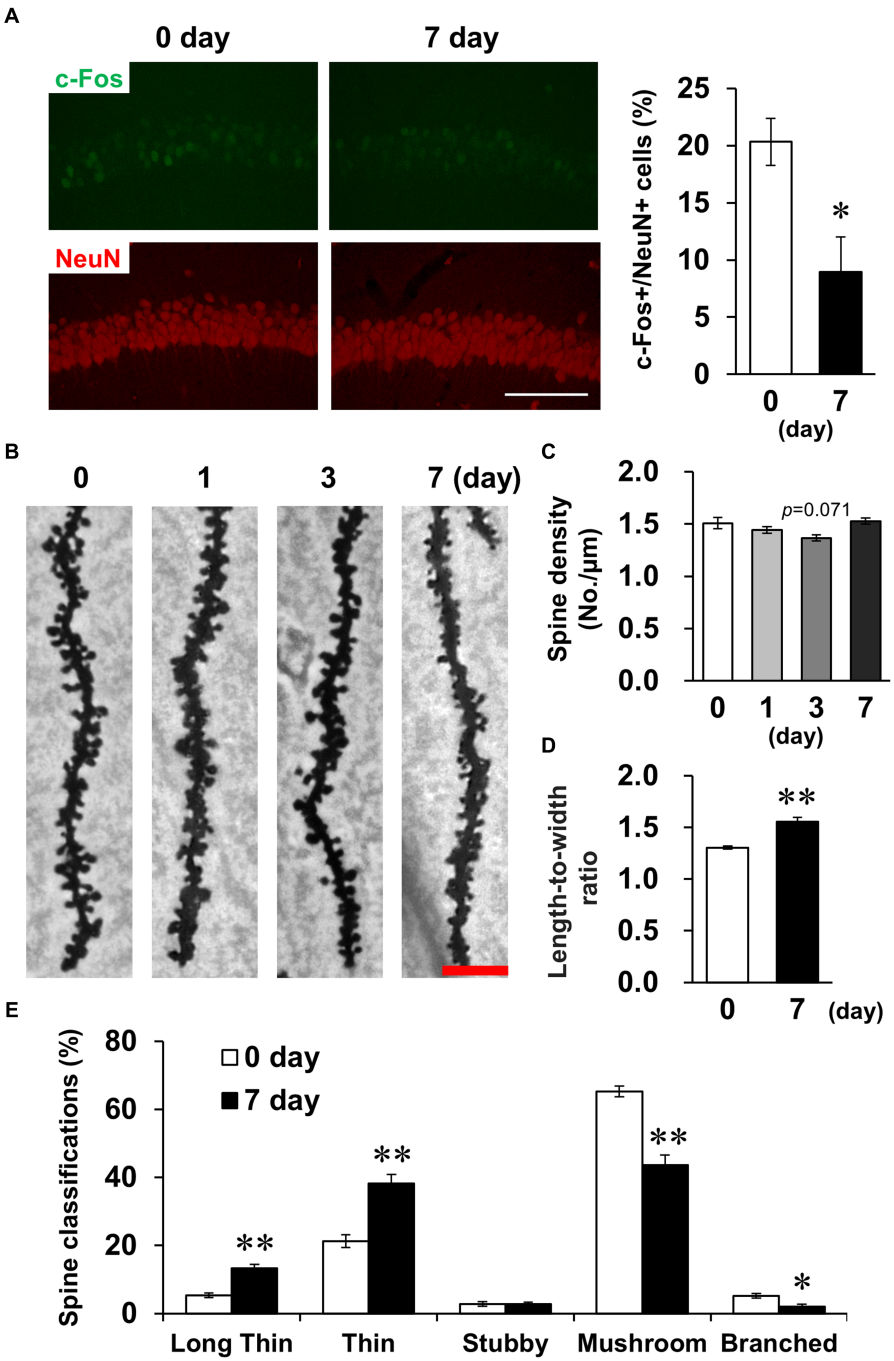
Effects of LPS on neuronal activity and dendritic spine properties. **(A)** Representative c-Fos (green) and NeuN (red)-stained images in the hippocampal CA1 regions of mice 0 and 7 days after LPS injection (3 mg/kg, *i.p.*, single dose, left) and percentage of c-Fos-positive cells in NeuN-positive cells (right). Scale bar = 100 μm. n = 6 mice/group. ^*^p < 0.05 (unpaired Student’s t-test). **(B)** Representative images in the hippocampal CA1 region stained with Golgi-Cox staining of mice 0–7 days after LPS injection. Scale bar = 5 μm. **(C)** Quantification of spine density (number of spines/μm) in the hippocampal CA1 region of mice 0–7 days after LPS injection. *n* = 6 mice/group. **(D,E)** Quantification of length-to-width ratio **(D)** and classification (%, **E**) of the spines by morphology in the hippocampal CA1 region of mice 0 and 7 days after LPS injection using the Reconstruct application. n = 6 mice/group. ^*^p < 0.05, ^**^p < 0.01 (unpaired Student’s *t*-test). All data are expressed as means ± S.E.M.

Subsequently, we examined whether LPS affected CA1 neuronal activity. The density of cells positive for c-Fos, a measure of neuronal activity, was significantly decreased on day 7 (Figure 5A). The density and morphology of dendritic spines, which are composed of synapses, affect neuronal circuits and plasticity, contributing to memory and learning (Runge et al., 2020). Golgi-Cox staining revealed that spine density did not change on day 7 (Figures 5B,C), but spine morphology was altered, which was characterized by elongated spines (Figure 5D). Because immature spines have an elongated morphology, spines were classified according to their morphology. The percentage of immature spines (long thin and thin) increased and the percentage of mature spines (mushroom and branched) decreased on day 7 (Figure 5E). Taken together, these findings demonstrate that LPS caused suppression of neuronal activity accompanied by increased immature CA1 dendritic spines on day 7.

### 3.4. LPS transiently activated glial cells, resulting in morphological changes

**Figure 6:**
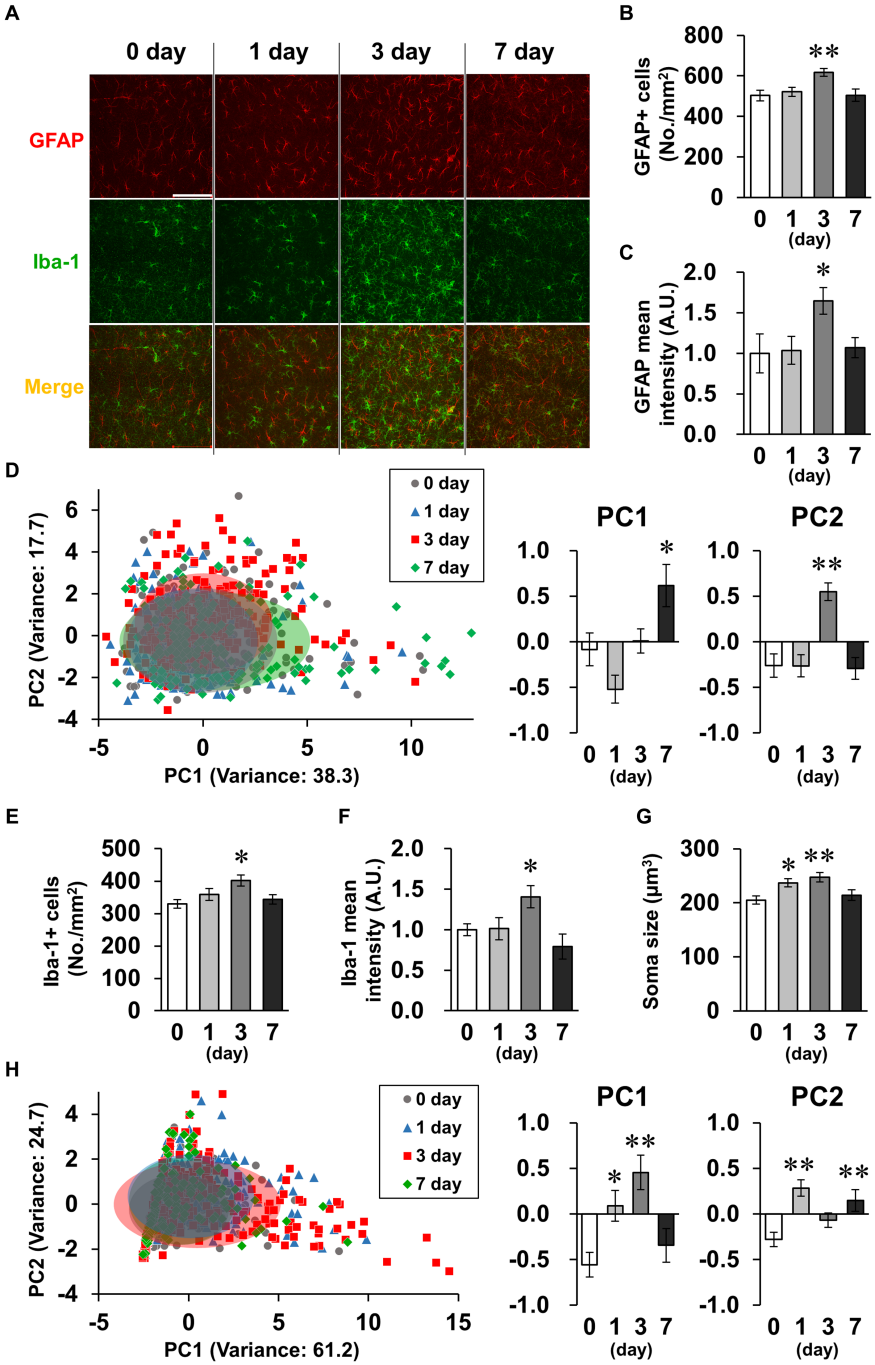
Effects of LPS on glial activation characterized by morphology. **(A)** Representative GFAP (red), Iba-1 (green), and DAPI (blue)-stained images in the hippocampal CA1 region of mice 0–7 days after LPS injection (3 mg/kg, *i.p.*, single dose). Scale bar = 100 μm. **(B,C)** Density of GFAP-positive cells (number of cells/mm^2^, **B**) and GFAP mean fluorescence intensity (A.U., **C**) in the hippocampal CA1 region of mice 0–7 days after LPS injection. *n* = 7 mice/group. **(D,H)** Principal component (PC) analysis reduced the dimensionality of the feature space consisting of 16 (astrocyte, **D**) or 6 (microglia, **H**) morphological features extracted from the images. A two-dimensional PC analysis plot representing the first two PCs, overlaid with ellipses corresponding to three standard deviations centered on the mean. Graphs show PC1 and PC2. n = 172–192 cells (from 7 mice)/group **(D)** and 118–279 cells (from 7 mice)/group **(H)**. **(E,F)** Density of Iba-1 positive cells (number of cells/mm^2^, **E**) and mean fluorescence intensity of Iba-1 (A.U., **F**) in the hippocampal CA1 region of mice 0–7 days after LPS injection. *n* = 7 mice/group. **(G)** Mean soma size (μm^3^) of Iba-1-positive cells in the hippocampal CA1 region of mice 0–7 days after LPS injection. *n* = 85–105 cells (from 7 mice)/group. ^*^p < 0.05, ^**^p < 0.01 vs. 0 day (Dunnett’s test). All data are expressed as means ± S.E.M.

Spine density tended to decrease transiently on day 3 (Figure 5C). Because activated glial cells potentially phagocytose spines, leading to decreased spine density (Stein and Zito, 2019), we subsequently investigated glial cell activation. GFAP and Iba-1, indicators of astrocyte and microglial activation, respectively, were detected by immunostaining (Figure 6A). In astrocytes, the density of GFAP-positive cells and mean GFAP fluorescence intensity increased on day 3 and recovered to baseline levels by day 7 (Figures 6B,C). Because astrocytic activation is strongly associated with morphological changes, we evaluated process morphology with the SMorph application, and performed PC analysis of 16 parameters (Supplementary Figure S2). The first two PCs described approximately 56% of the observed variability in the data (Figure 6D). PC1 was increased on day 7, which significantly correlated with projected area and total branch length, and PC2 was increased on day 3, which was significantly correlated with average length of terminal branches and average branch width (Supplementary Figure S4A). According to these parameters, process widths and lengths increased on day 3, and process number and total length increased on day 7. In microglia, like in astrocytes, the density of Iba-1-positive cells and the mean fluorescence intensity of Iba-1 increased on day 3 and recovered to baseline levels on day 7 (Figures 6E,F), and the soma size of microglia increased significantly on days 1 and 3, which is characteristic of activated microglia (Figure 6G). We also performed PC analysis using six parameters for microglia (Supplementary Figure S3). The first two PCs accounted for approximately 85% of the observed variability in the data (Figure 6H). PC1 was increased on days 1 and 3, which was significantly correlated with the number of branches and junctions, and PC2 was increased on days 1 and 7, which was significantly correlated with average and maximum length of branches (Supplementary Figure S4B). According to these parameters, the number of processes increased on day 3, and process length significantly increased on days 1–7 in microglia (Supplementary Figure S3).

### 3.5. LPS transiently increased microglial inflammatory factors and phagocytosis

**Figure 7:**
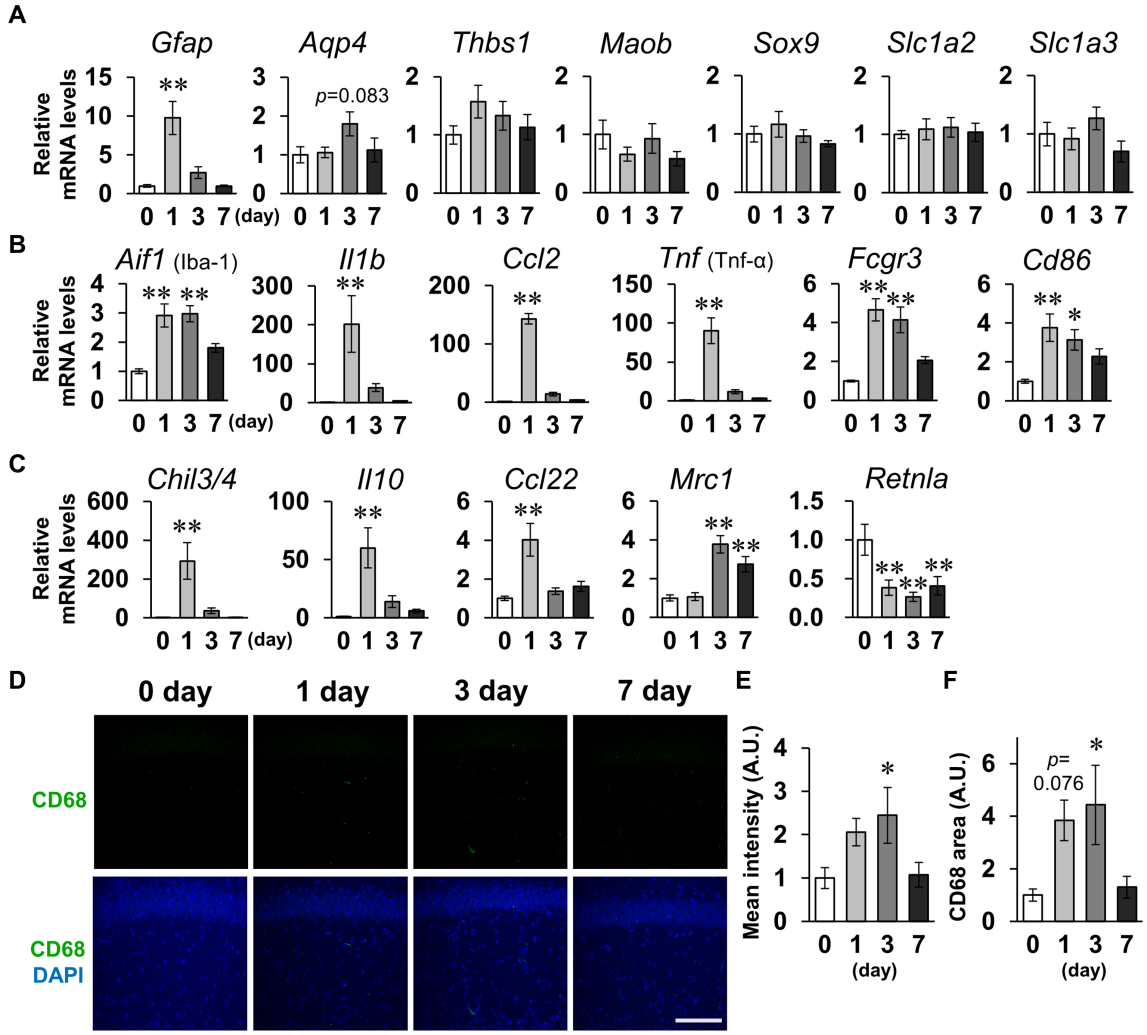
Effects of LPS on expression of glial activation markers. **(A–C)** Whole hippocampus mRNA levels of the astrocytic activation markers *Gfap, Aqp4, Thbs1, Maob, Sox9, Slc1a2,* and *Slc1a3* **(A)**; microglial M1 markers *Aif1, Il1b, Xcl2, Tnf, Fcgr3,* and *Cd86* **(B)**; and M2 markers *Chil3/4, Il10, Ccl22, Mrc1,* and *Retnla* **(C)** of mice 0–7 days after LPS injection (3 mg/kg, *i.p.*, single dose). n = 8 mice/group. **(D)** Representative CD68 (green) and DAPI (blue)-stained images in the hippocampal CA1 region of mice 0–7 days after LPS injection. Scale bar = 100 μm. **(E,F)** Mean fluorescence intensity **(E)** and area **(F)** of CD68. *n* = 8 mice/group. ^*^p < 0.05, ^**^p < 0.01 vs. 0 days (Dunnett’s test). All data are expressed as means ± S.E.M.

Subsequently, we measured mRNA levels in the hippocampus for several factors considered to be indicators of glial cell activation (Ikeshima-Kataoka, 2016; Jurga et al., 2020; Escartin et al., 2021; Guo et al., 2022). For the seven factors (*Gfap*, *Aqp4*, *Thbs1*, *Maob*, *Sox9*, *Slc1a2*, and *Slc1a3*) that are indicators of reactive astrocytes, RT-qPCR analysis revealed that only *Gfap* significantly increased on day 1, and *Aqp4* non-significantly increased on day 3 (Figure 7A). No significant differences between groups were detected for any of the astrocytic activation factors on day 7. For the six microglial M1 markers (*Aif1*, *Il1b*, *Ccl2*, *Tnf*, *Fcgr3*, and *Cd86*), there were significant increases in all the factors on day 1 and no factors significantly differed between groups on day 7 (Figure 7B). The M1 markers *Aifi*, *Fcgr3,* and *Cd86* were also significantly increased on day 3. For the five M2 markers, four factors (*Chil3/4*, *Il10*, *Ccl22*, and *Mrc1*) were significantly increased on days 1 or 3 (Figure 7C). *Mrc1* remained significantly increased on day 7. Contrastingly, *Retnla* significantly decreased on days 1, 3, and 7. These data suggest that the robust LPS-induced inflammation peaked on days 1–3 and approximately recovered to baseline levels by day 7. The decrease in dendritic spine density and glial cell inflammation were both maximal on days 1–3. We thus hypothesized that activated microglia phagocytosed the spines, and performed immunostaining for CD68, the microglia-specific phagocytic marker (Figure 7D). CD68-positive cells did not express the astrocyte marker GFAP or the neuronal marker NeuN (Supplementary Figure S5). Mean CD68 fluorescence intensity and area of CD68 increased significantly on day 3 (Figures 7E,F). It is likely that microglia phagocytosed spines on days 1–3, resulting in an increase in immature spines on day 7.

### 3.6. LPS induced different inflammatory responses depending on brain region

**Figure 8:**
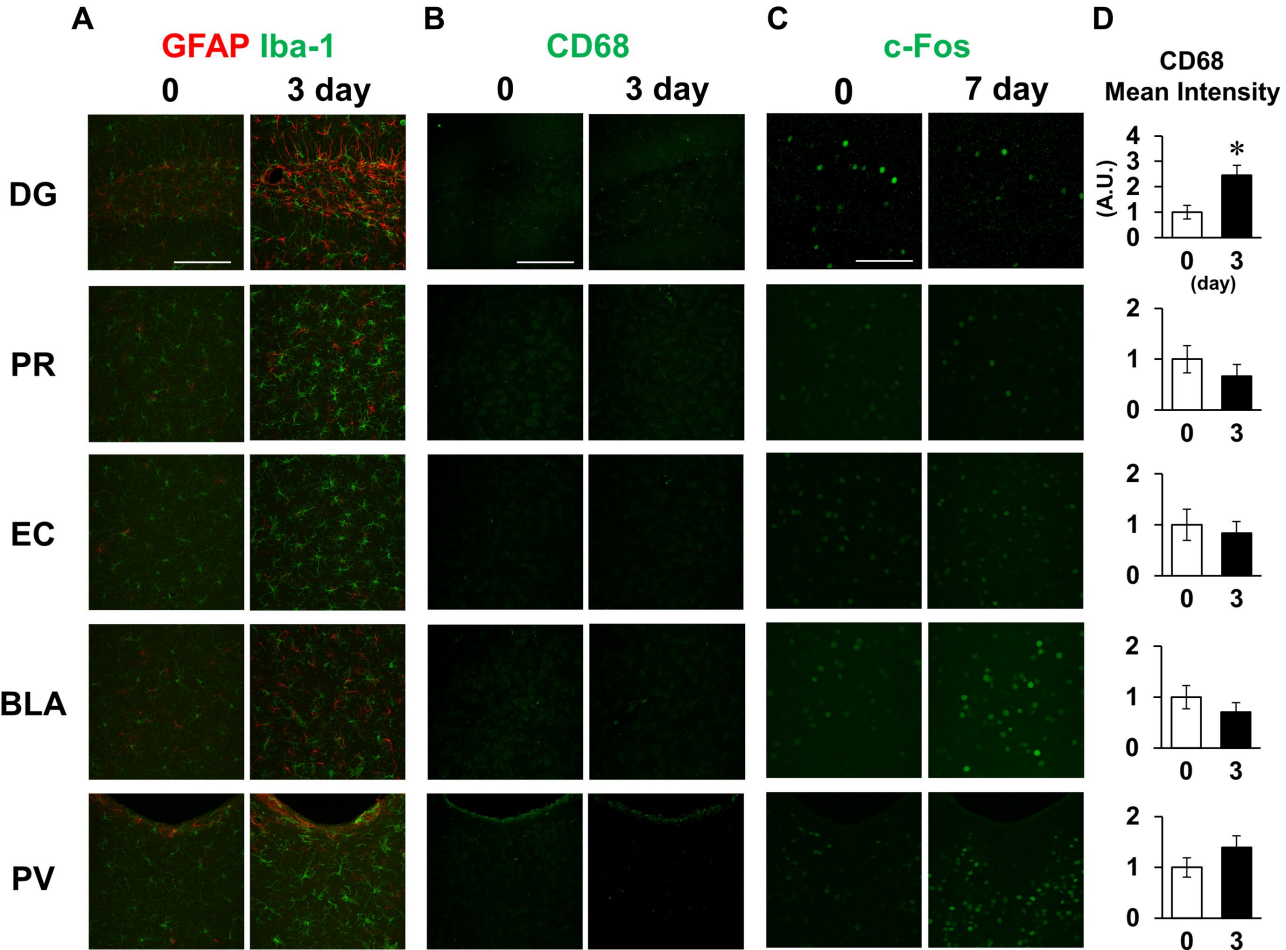
Effect of LPS on neuronal and glial activation in different brain regions. **(A,B)** Representative GFAP and Iba-1 **(A)**, and CD68 **(B)** images in the dentate gyrus (DG), perirhinal cortex (PR), entorhinal cortex (EC), basolateral amygdala (BLA), and paraventricular hypothalamic nucleus (PV) of mice 0 and 3 days after LPS injection (3 mg/kg, *i.p.*, single dose). **(C)** Representative c-Fos images in the specified brain regions of mice 0 and 7 days after LPS injection. **(D)** Mean fluorescence intensity of CD68 (A.U.) in each brain region of mice 0 and 3 days after LPS injection. n = 5 mice/group. ^*^p < 0.05 (unpaired Student’s t-test). All data are expressed as means ± S.E.M.

Finally, we also examined the inflammatory responses to LPS in other brain regions, including the dentate gyrus, perirhinal cortex, entorhinal cortex, basolateral amygdala, and paraventricular hypothalamic nucleus, which include the regions involved in short-term memory. Glial cells were activated in all regions examined on day 3 (Figure 8A). Meanwhile, CD68 fluorescence intensity was increased in the hippocampal dentate gyrus without significant changes in the other regions on day 3 (Figures 8B,D). c-Fos expression was decreased in the hippocampal dentate gyrus on day 7, while no difference was observed in the perirhinal cortex or entorhinal cortex (Figure 8C). Contrastingly, LPS increased c-fos expression in the basolateral amygdala and paraventricular nucleus (Figure 8C). These results suggest that glial cell activation occurred throughout the brain, but that there were regional differences in microglial phagocytosis and neuronal activity in response to LPS challenge.

## 4. Discussion

In the present study, we identified that 7 days after LPS injection, mice with recovery of systemic symptoms exhibited short-term memory impairment in behavioral tests. Suppression of hippocampal CA1 neuronal activity and decreased synaptic strength due to immature spines likely contribute to short-term memory impairment in this context. Decreased spine density due to enhanced phagocytosis of activated microglia could be responsible for increased immature spines (see Graphical abstract).

At the commonly used dose of 1 mg/kg LPS, the behavioral testing parameters for depression, anxiety, and anhedonia were affected in experiments conducted within 24 h following LPS injection, consistent with previous studies (Moraes et al., 2017; Yu et al., 2021; Song et al., 2022). However, significant systemic symptoms following LPS challenge were observed, with decreased spontaneous activity, body weight, and water intake. Therefore, it is possible that indicators of depression in previously reported behavioral tests, especially freezing and immobility time, are due in part to systemic symptoms. We also suggest that the apparent decrease in sucrose preference is due to decreased total water intake, as distilled water intake did not change.

Under acute pathological conditions evoked by LPS, the inflammatory cytokine CCL2 secreted by blood vessel cells promotes excitatory synaptic transmission in glutamatergic neurons of the hippocampus (Duan et al., 2018). α1-Adrenoceptor-induced downregulation of membrane GluR1 is involved in LPS-induced depressive-like behavior (Sekio and Seki, 2015). Unlike these acute responses, the transient decrease in spines followed by the increase in immature spines, as observed in the present study, required several days, indicating distinct pathogenesis from the acute response.

The LPS dose of 3 mg/kg used in the present study produced LPS blood levels similar to those in patients with sepsis (Casey et al., 1993). At this dose of LPS, BBB permeability was not affected, and none of the mice died within 7 days following administration of this dose. Meanwhile, the BBB was disrupted in mice 18 h after high-dose LPS injection (18 mg/kg, *i.p*.) (Kikuchi et al., 2019). Further, administration of 10 mg/kg LPS *i.p*. causes 100% mortality within 72 h (Ahn et al., 2017). Therefore, the LPS doses required to induce BBB disruption result in a high mortality rate in mice, while the dose used in the present study (3 mg/kg) did not cause mortality with 7 days of injection, and acute systemic effects were mild.

We evaluated the pathogenesis of the LPS inflammatory response, focusing primarily on the hippocampus, as in this context LPS affected behaviors associated with short-term memory deficits, as identified by NORT and OLRT testing. OLRT is a primarily hippocampus-dependent test, while NORT is dependent on multiple brain regions, but is not affected by hippocampal injury or inactivation in mice (Barker and Warburton, 2011; Denninger et al., 2018). Contrary to these reports, some studies have reported that NORT is affected by the hippocampus (Broadbent et al., 2010; Cohen and Stackman, 2015; Cinalli et al., 2020). Involvement of the hippocampus in NORT is likely to depend on the latency between the training and test sections of a study. At short latencies, only the perirhinal cortex and entorhinal cortex are involved, while for latencies >10 min, the hippocampus is also involved (Cohen and Stackman, 2015). In the present study, OLRT and NORT exploration times were decreased in LPS-injected mice subjected to conditioning with long latency periods (24 h), suggesting that the hippocampus was involved in the short-term memory impairment detected in these tests. Meanwhile, in the present study, it was not possible to identify which memory processes were impaired, for example, formation, maintenance, or recall of memory (Josselyn and Tonegawa, 2020). Changes in dendritic spine morphology and number are involved in memory formation (Basu and Lamprecht, 2018). Further mechanisms of memory impairment need to be elucidated by measuring neuronal activity before and after training and testing sections.

Seven days after LPS injection, glial cell activation had returned nearly to baseline levels, together with recovery of systemic symptoms. Meanwhile, some parameters remained altered in both astrocytes and microglia, including both the morphology and expressions of glial activation markers 7 days after LPS injection. It is thus possible that LPS subsequently caused longer-duration changes in glial cell properties. Especially, the processes of both astrocytes and microglia remained elongated 7 days after LPS injection. The processes are likely to remain longer to be more sensitive to abnormalities, serving as an alert system. This is consistent with the two-hit hypothesis in mental disorders (Cao et al., 2021). When microglia are activated by inflammation in early life, the threshold for activation is lowered, pathologically increasing microglial sensitivity to stress in later life. Because the present study injected LPS into adult mice, brain pathology should be investigated over a longer period after LPS injection in future studies.

Furthermore, changes in the morphology (activation) of glial cells could be responsible for short-term memory impairment. Astrocyte process elongation interferes physically with spine maturation and expansion, impairing memory formation (Zorec et al., 2015). Moreover, fine astrocyte and microglial processes cover synapses. Astrocytes affect neuronal plasticity via the uptake and release of glutamate and D-serine, while microglia release inflammatory cytokines, such as TNF-α, IL-6, and IL-1β, which affect neuronal excitability by modulating the activities of many classes of voltage-gated channels and receptors (Sancho et al., 2021). Similar to these findings, in the present study, increased coverage of the synapse via glial process elongation could affect neuronal activity and plasticity.

The time course of decreased spine density coincided with that of increased CD68, and increased CD68 concomitant with decreased density of c-Fos positive cells occurred in the hippocampus. We thus postulate that M1-like phagocytic microglia phagocytosed the spines. Meanwhile, there are microglia-independent mechanisms in spine disappearance, such as astrocytic phagocytosis and spine retraction accomplished in only neurons (not involving other cells) (Stein and Zito, 2019). We have not identified whether the pathology observed in the present study is the cause of short-term memory impairment. It is necessary to investigate whether inhibition of microglial phagocytosis prevents spine morphological abnormalities and short-term memory impairment.

The mechanism by which glial cells are activated by *i.p.* LPS injection remains unclear. Multiple regulatory mechanisms could be responsible for LPS-induced hippocampal gliosis. First, LPS in the blood crosses the BBB and enters the brain parenchyma, activating TLR4 in glial cells (Vargas-Caraveo et al., 2017). Second, damage-associated molecular patterns (DAMPs) generated by neurons in response to heat, stress, or excitotoxicity activate TLR4 in glial cells (Afridi and Suk, 2023). Third, increased blood cytokines activate vascular endothelial cells and release inflammatory factors into the brain parenchyma (Bowyer et al., 2020). TLR4-deficient mice are hyporesponsive to LPS (Hoshino et al., 1999). LPS injection increased brain endotoxin level in the present study, suggesting that LPS did cross BBB. Therefore, in our system, LPS was likely to directly activate TLR4 expressed on microglia and/or astrocytes. Although LPS is not thought to pass through the intact BBB (Banks and Robinson, 2010), a conflicting study reported that LPS in fact enters the brain in physiological conditions *via* a lipoprotein-mediated transport mechanism (Vargas-Caraveo et al., 2017). Further investigation is needed to assess the contribution of these processes.

LPS caused different responses depending on brain region. Glial cell activation was detected in all regions examined, but regional differences were observed in neuronal activity and phagocytotic phenotype of microglia. Notably, LPS suppressed neuronal activity only in the hippocampus. Consistent with the present study, the limbic system including the hippocampus is more susceptible to inflammation in mouse models of inflammatory diseases of peripheral organs such as colitis, gastritis, and cystitis (Sun et al., 2022). Furthermore, the hippocampus is more vulnerable to stress, ischemia, trauma, and aging than other brain regions (Bartsch and Wulff, 2015). Although several hypotheses have been proposed, it is unclear that the mechanism underlying increased susceptibility to inflammation in specific brain regions (Sun et al., 2022). Interestingly, there were regional differences in the microglial phagocytosis, even though morphological activation of microglia occurred in all regions examined in the present study. Elucidation of the mechanisms of these regional differences can provide novel therapeutic targets. In addition, consistent with the present study, *i.p.* LPS injection has previously been reported to increase neuronal activation in the paraventricular nucleus and amygdala (Araki et al., 2016). Activation of the paraventricular nucleus increases blood corticosterone levels *via* corticotropin-releasing hormone, and corticosterone activates the amygdala. LPS injection increases blood corticosterone levels (Haskó et al., 1995), suggesting potential involvement of these pathways. In the present study, limited types of behavioral tests were performed, and several brain regions were observed. Therefore, we cannot deny that there are no brain abnormalities other than hippocampus-dependent short-term memory impairment following recovery from systemic symptoms caused by LPS.

Finally, the elucidation of pathogenic mechanisms in rodent models is an urgent issue, with the goal of clinical application to the treatment of mental disorders caused by peripheral inflammatory diseases. Cell types, receptors, and factors associated with the identified mechanisms can be potential therapeutic targets. The pathological evaluation following recovery from systemic and acute symptoms, which was the focus of the present study, provides novel perspectives for studies using the LPS-induced inflammation model.

## Data availability statement

The raw data supporting the conclusions of this article will be made available by the authors, without undue reservation.

## Ethics statement

The animal study was approved by Committee on Animal Experimentation, Faculty of Veterinary Medicine, Hokkaido University. The study was conducted in accordance with the local legislation and institutional requirements.

## Author contributions

KM: Conceptualization, Data curation, Formal analysis, Funding acquisition, Investigation, Methodology, Project administration, Visualization, Writing – original draft, Writing – review & editing. SW: Data curation, Formal analysis, Funding acquisition, Investigation, Methodology, Software, Visualization, Writing – review & editing. RE: Funding acquisition, Methodology, Visualization, Writing – review & editing. TK: Conceptualization, Methodology, Visualization, Writing – review & editing. KO: Funding acquisition, Methodology, Project administration, Supervision, Visualization, Writing – review & editing.

## Footnotes

^1^https://synapseweb.clm.utexas.edu/
