# Supplementary material for: Short-term memory impairment following recovery from systemic inflammation induced by lipopolysaccharide in mice
Source: Front Neurosci. 2023 Oct 18;17:1273039. doi: 10.3389/fnins.2023.1273039 (PMC10618367; doi:10.3389/fnins.2023.1273039)
Supplement: Supplementary file 1 [file Data_Sheet_1.DOCX]

Supplementary Material

**Supplementary Table 1. Antibodies used for immunohistochemistry**

| Target protein | Dilution | Host animal | Catalog number | Supplier |
| --- | --- | --- | --- | --- |
| NeuN | 1:200 | Mouse | MAB377 | Sigma-Aldrich,  MO, USA |
| c-Fos | 1:3000 | Rabbit | ab190289 | abcam,  Cambridge, UK |
| GFAP | 1:200 | Mouse | 11051 | Immuno-Viological Laboratories, Gunma, Japan |
| Iba-1 | 1:1000 | Rabbit | 019-19741 | FUJIFILM Wako,  Osaka, Japan |
| CD68 | 1:500 | Rabbit | ab125212 | abcam,  Cambridge, UK |

| Gene  nama | Gene symbol | Product  size (bp) | Sequence (5'->3')  (upper: forward; lower: reverse) |
| --- | --- | --- | --- |
| *glial fibrillary acidic protein* | *Gfap* | 244 | CACCAAACTGGCTGATGTCTAC |
|  |  |  | AACCTTTCTCTCCAAATCCACAC |
| *aquaporin 4* | *Aqp4* | 228 | GCTGTGATTCCAAACGAACTGATG |
|  |  |  | AGAAGACATACTCATAAAGGGCACC |
| *thrombospondin 1* | *Thbs1* | 160 | CTGGACTTGCTGTAGGTTATGATG |
|  |  |  | TAGGACTGGGTGACTTGTTTCC |
| *monoamine oxidase B* | *Maob* | 143 | CCAAAGAAGAAAGACTGAGGAAAC |
|  |  |  | AGGGAAGTAGGTTGTGTAGCAG |
| *SRY (sex determining region Y)-box 9* | *Sox9* | 117 | CAAGAAAGACCACCCCGATTAC |
|  |  |  | AAGATAGCATTAGGAGAGATGTGAG |
| *solute carrier family 1 (glial high affinity glutamate transporter), member 2* | *Slc1a2* | 113 | GGAAGAAGAACGACGAGGTGTC |
|  |  |  | CTTTGTCACTGTCTGAATCTGC |
| *solute carrier family 1 (glial high affinity glutamate transporter), member 3* | *Slc1a3* | 216 | AGGGAAGATTGTTGAGATGGAAGA |
|  |  |  | GGTAGGGTGGCAGAACTTGAGG |
| *allograft inflammatory factor 1* | *Aif1* | 94 | CTGCCCTGATTGGAGGTGGATG |
|  |  |  | CTCTGGCTCACGACTGTTTCTTTTT |
| *interleukin 1 beta* | *Il1b* | 148 | AGTTGACGGACCCCAAAAGA |
|  |  |  | CAGCTTCTCCACAGCCACAA |
| *C-C motif chemokine ligand 2* | *Ccl2* | 169 | CTCTCTCTTCCTCCACCACCAT |
|  |  |  | CTCTCCAGCCTACTCATTGGGA |
| *tumor necrosis factor* | *Tnf* | 146 | CCCCTTTACTCTGACCCCTTTATTG |
|  |  |  | ACTGTCCCAGCATCTTGTGTTTC |
| *Fc receptor, IgG, low affinity III* | *Fcgr3* | 184 | ATTTCTCTATCCCAAAAGCCAACCA |
|  |  |  | GCAAACAGGAGGCACATCACTA |
| *CD86 antigen* | *CD86* | 240 | GAGGAAGAAAGAGGAGCAAGCAG |
|  |  |  | TTGTAAATGGGCACGGCAGATA |
| *chitinase-like 3/4* | *Chil3/4* | 192 | CTGAATGAAGGAGCCACTGAG |
|  |  |  | GTGACAGAAAGAACCACTGAAG |
| *interleukin 10* | *Il10* | 175 | GTGAAAATAAGAGCAAGGCAGTGG |
|  |  |  | GTCCAGCAGACTCAATACACAC |
| *C-C motif chemokine ligand 22* | *Ccl22* | 173 | GTCTTTTACTTTCTCTGCCCCAC |
|  |  |  | ACTCTCCAGTTCGTCTATTTGGC |
| *mannose receptor, C type 1* | *Mrc1* | 144 | CTGACTGTGTAGTTGTGATTGGTG |
|  |  |  | CATCTTTTGGAGTAGTGGTTGGAG |
| *resistin like alpha* | *Retnla* | 211 | CGTGGAGAATAAGGTCAAGGAAC |
|  |  |  | AACGAGTAAGCACAGGCAGTTG |
| *glyceraldehyde-3-phosphate dehydrogenase* | *Gapdh* | 74 | GCAAGAGAGAGGCCCTCAG |
|  |  |  | TGTGAGGGAGATGCTCAGTG |

**Supplementary Table 2. Primer sequences used for RT-qPCR**


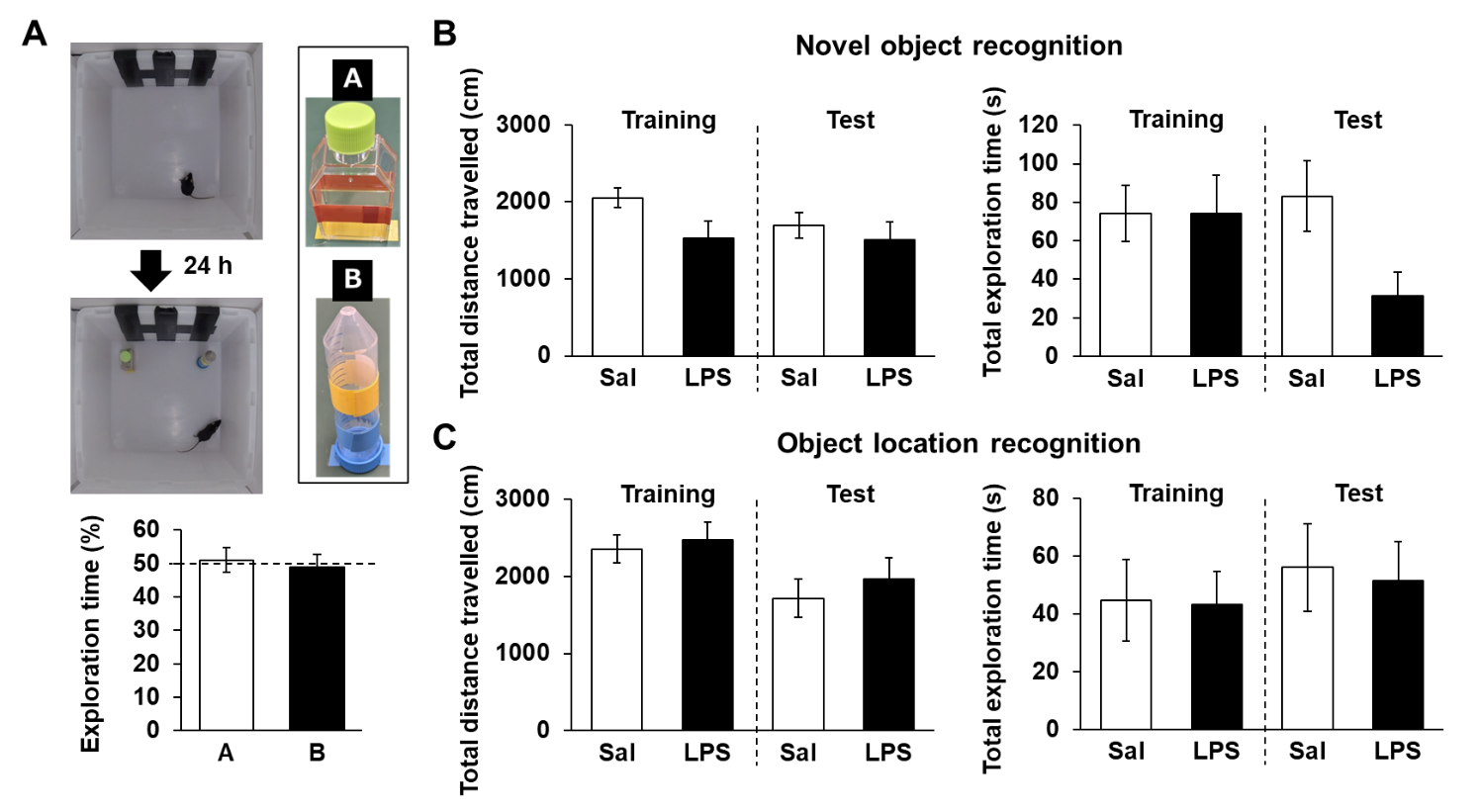


**Supplementary Figure 1. Effects of LPS on novel object recognition and object location recognition tests**

**(A)** The test section was conducted 24 hours after the habituation section in normal mice. **(B, C)** Total distance traveled (cm) and total exploration time (s) in novel object recognition tests **(B)** and object location recognition tests **(C)** with mice 6 days (training section) or 7 days (test section) after LPS injection (3 mg/kg*, i.p.,* single dose). n = 9–10 mice/group.

**
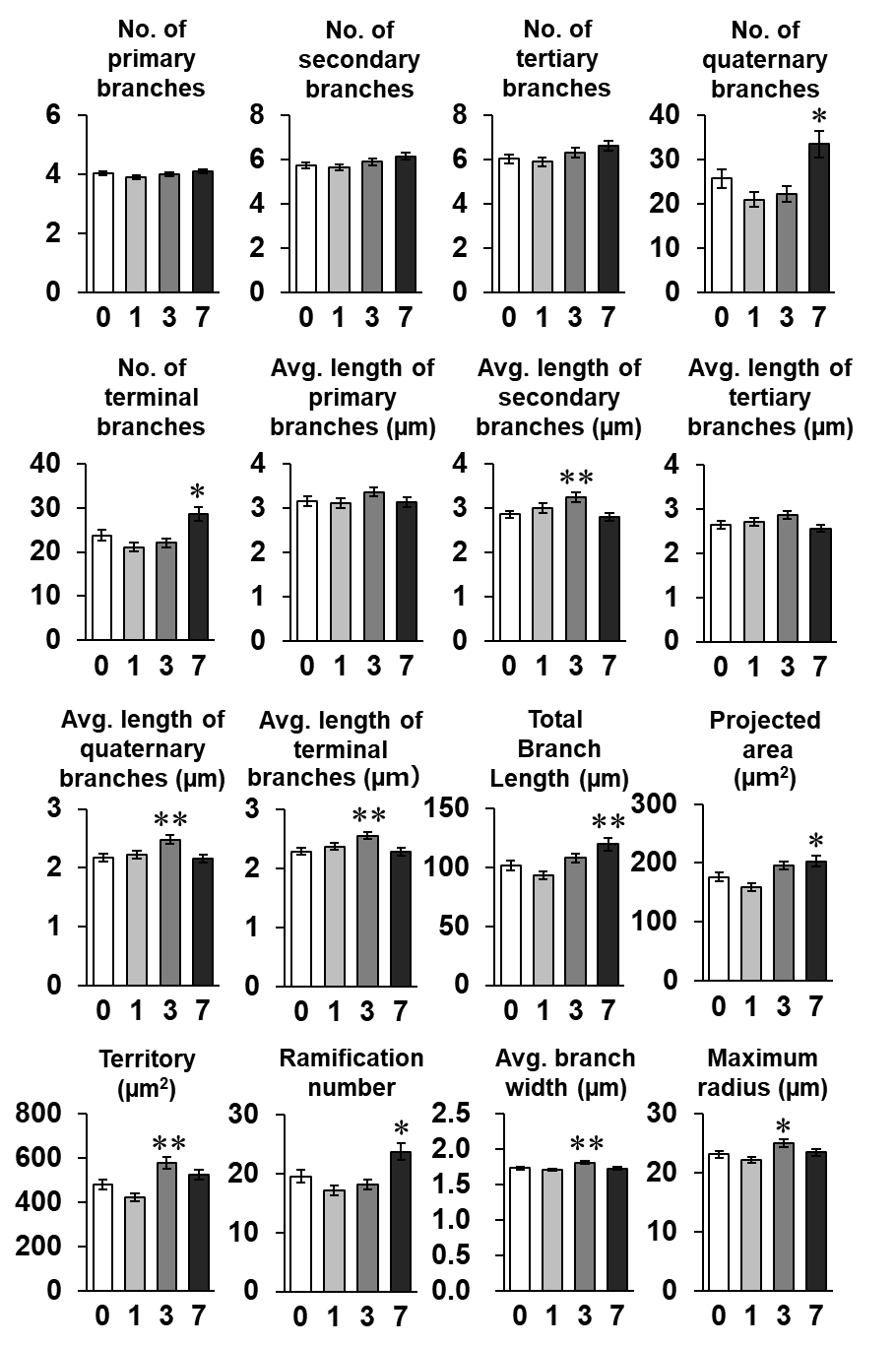
**

**Supplementary Figure 2. Effect of LPS on astrocytic morphology**

Parameters of astrocytic morphology were analyzed with SMorph. **p* < 0.05, **p < 0.01 vs. 0 day (Dunnett’s test), n = 172–192 cells (from 7 mice)/grop. All data are presented as means ± S.E.M.


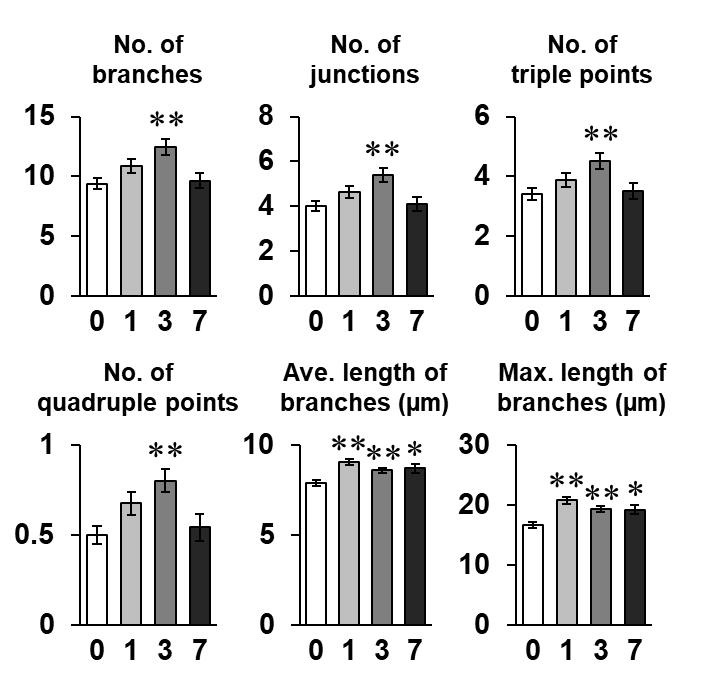


**Supplementary Figure 3. Effect of LPS on microglial morphology**

Parameters of microglial morphology analyzed with ImageJ. **p* < 0.05, **p < 0.01 vs. 0 day (Dunnett’s test), n = 118–279 cells (from 7 mice)/grop. All data are presented as means ± S.E.M.


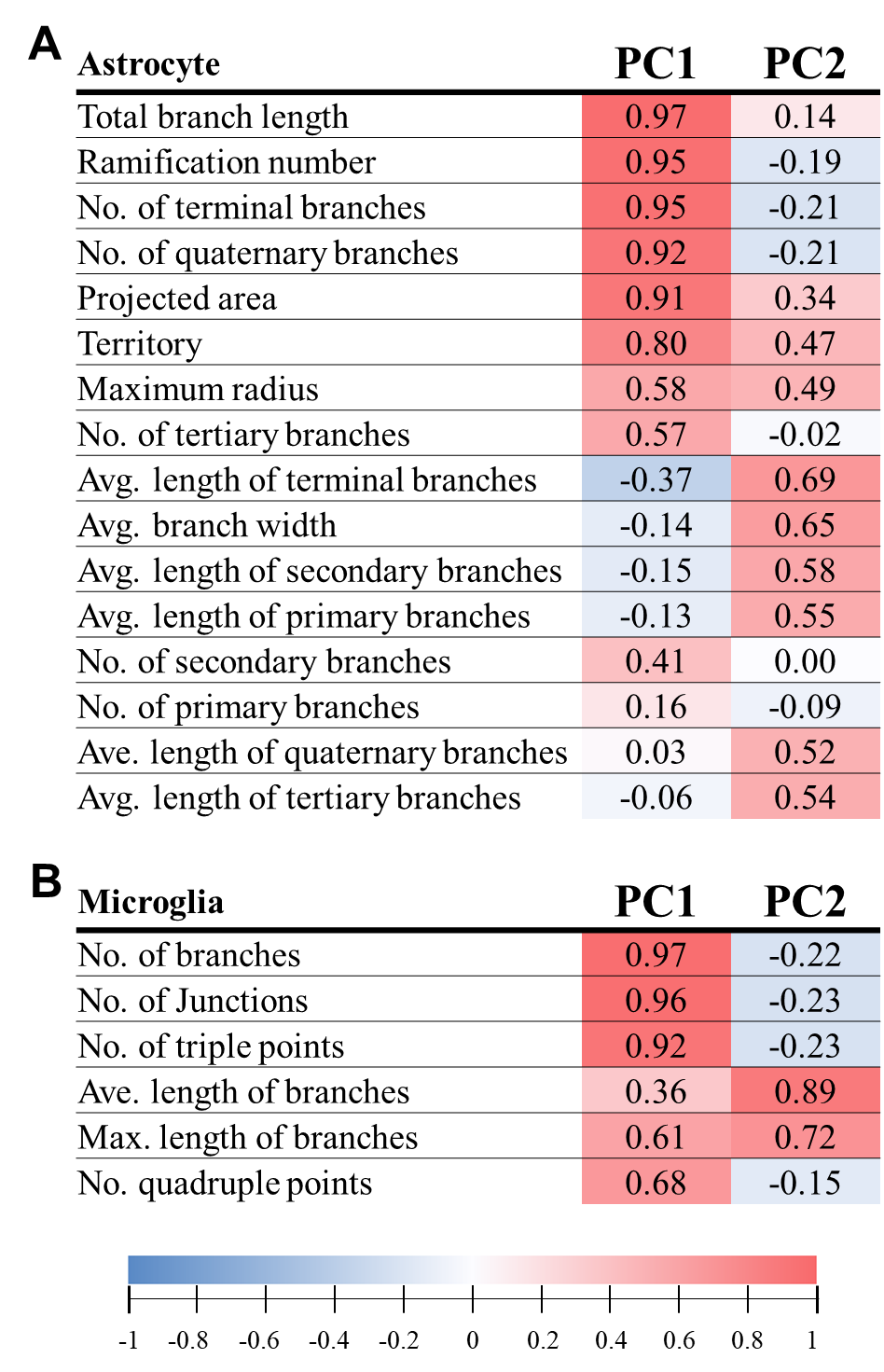


**Supplementary Figure 4. Contributions of each parameter to PC analysis**

Contributions of each parameter to principal components (PCs) in main Figures 6D, E. PC analysis decreased the dimensionality of the feature space consisting of 16 (astrocyte, **A**) or six (microglia, **B**) morphological features extracted from images.

**
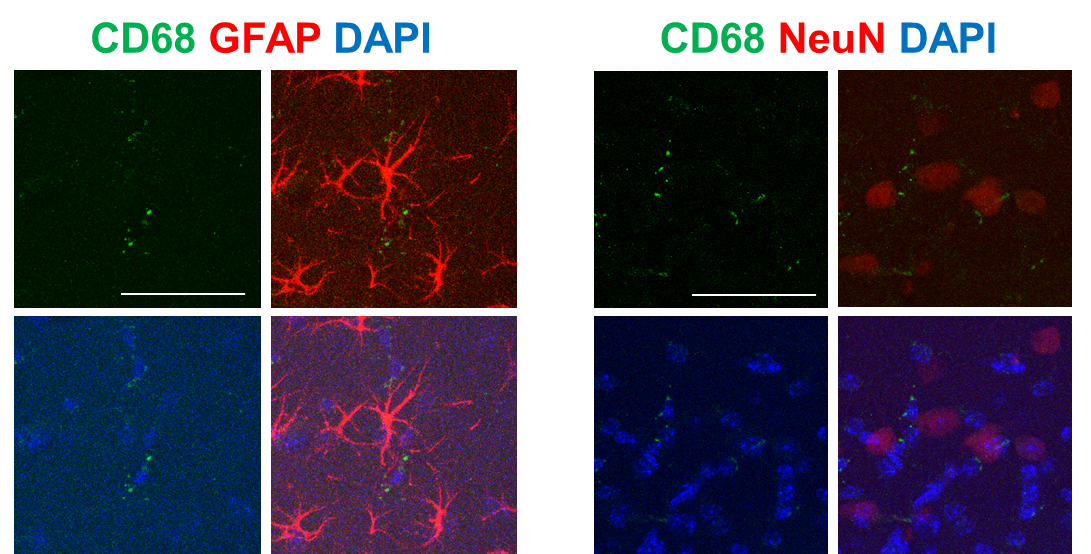
**

**Supplementary Figure 5. Cell specificity of CD68 staining**

Representative CD68 (green) and GFAP or NeuN (red)-stained images in the hippocampal CA1 region of mice 3 days after LPS injection. Scale bar = 100 µm.
